# Multi-objective predictive control based on the cutting tobacco outlet moisture priority

**DOI:** 10.1038/s41598-022-26694-x

**Published:** 2023-01-05

**Authors:** Zhiping Fan, Zhengyun Ren, Angang Chen

**Affiliations:** 1 School of Electrical and Electronic Engineering, Anhui Science and Technology University, Bengbu, 233000 China; 2 College of Information Science and Technology, Donghua University, Shanghai, 201620 China; 3 Guotai Junan Securities Co., Ltd., Shanghai, 200041 China

**Keywords:** Engineering, Chemical engineering, Mechanical engineering, Mathematics and computing, Computational science, Scientific data

## Abstract

In this paper, we propose a new priority multi-objective optimization strategy of system output variables in cutting tobacco process. The proposed strategy focuses on the cutting tobacco moisture-controlled output variables optimization in feasible regions with two levels according to the priority. This study aims to provide a novel technical support for the chemical industry contained drying process. In order to alleviate the lack of degree of freedom of the system, strict set-point control is given, meanwhile, other output variables adopt zone control. Firstly, the system control output variables are optimized in ascending order of priority. Secondly, the specific lower-level target constraints are first relaxed. Finally, the relaxation of other high-priority target constraints is stopped when the optimization is feasible. Thus, the system control output variables move along the optimal target trajectory. From the perspective of practical application of engineering, under the condition of disturbance existing in the cutting tobacco drying process, the simulation shows that the proposed approach has good robustness when there is disturbance, and the previous method cannot meet the control requirement. The proposed strategy meanwhile has better tracking effect through single and multiple output variables simulation, which compared with traditional predictive control in real cutting tobacco drying process.

## Introduction

Industrial drying is a preservation method applied with the aim of reducing the moisture content of products by using heat energy. Drying is a complex and polytropic process that involves coupled heat, mass, and momentum exchange in the drying medium. The drying process can be realized by different types of dryers, such as belt, conveyor, drum, fluidized bed, vacuum, rotary and spray, and their size, shape, and drying quantity are different, but the drying mechanism is similar.

Due to the different characteristics of substances to be dried (such as moisture content, bulk density, consistency, etc.), for each substance, a specific drying technology for intermittent or continuous drying was used in a specific dryer. The advanced process control strategy not only improves the quality of drying products, but also increases the product yield. The optimization strategy of the industrial drying process is implemented based on a drying mathematical model. In the regulation problem, the predicted value of the model is used to generate optimal control. In the estimation problem, the predicted value of the model and the real industrial measurement data are used to produce optimal state estimation. Therefore, the study of the drying mathematical model is essential to the optimization strategy.

The modelling methods include first-principle modelling (mechanism modelling) and empirical modelling (data modelling). In this paper, a mathematical model of the tobacco drying process is established. Tobacco is a very complex biomass substrate, and redrying is a transitional stage between tobacco product processing and cigarette production. The drying process of cutting tobacco adopts a combination drying technique of conductive drying and forced convection to remove unbound free moisture from the surface of cutting tobacco, and then remove combined moisture from the inside of cutting tobacco^1,2^ to meet the technological requirement of subsequent cigarette production. At the same time, the nutrients and fragrance in cutting tobacco are retained^3^.

The advantage of first-principles modelling is that it can build highly complex process models and establish accurate nonlinear models. First-principle models are the preferred modelling strategy in industry for control objectives with strict production requirements and the needs for model portability and scalability^4,5^. The cutting tobacco drying process mainly involves two stages, the constant rate evaporation period and the decreasing rate evaporation period after the preheating period^6^. During the constant rate evaporation period, evaporation occurs on the outer surface of the cutting tobacco to remove unbound water (free water) from the surface of the cutting tobacco, and the constant rate evaporation period ends at the critical moisture content. Then, the rate of the evaporation period begins to decline until the required final moisture content is reached. During descent rate evaporation, the drying rate decreases because the moisture inside the cutting tobacco is slowly transported to the surface through the gradually increasing temperature of the cutting tobacco before evaporating from the surface^7–9^. The amount of moisture removed during the drop rate is small, but the time spent is quite long. Therefore, the implementation of a process control strategy was needed to improve the drying rate of the whole drying process to obtain the required tobacco outlet moisture. The cutting tobacco drying process is the most critical process in cigarette production. The main function of cutting tobacco drying machines is to control the moisture content of the cutting tobacco in a certain range to meet the technological requirements. The cutting tobacco drying process is a complex, dynamic, highly nonlinear, strongly interactive, continuously correlated, multivariate heat transfer process, which also has transient coupled momentum, heat and mass transfer, and time-varying physicochemical and structural changes of dried products^10^. The mathematical modelling of the drying process is the basis of drying process strategy research, which is very important to optimize and improve the running state and performance of the drying process.

Model Predictive Control (MPC) is a well-known method which has been broadly used in real industrial as an effective way of dealing with multi-variables constrained control problem. MPC depends on predictive models, which is to get the control signal by solving open-loop finite-horizon optimal control problem at every sample time. Due to MPC cannot solve the model uncertainties and disturbance, many modified MPC is proposed to satisfy the stability and anti-disturbance performance. Zhang et al.^11^ combine linear extended state observer with fuzzy MPC to solve the disturbance rejection ability problem by estimating and compensating. Wu et al.^12^ developed a T-S fuzzy stable model PC tracking controller to realize the aim of offset-free tracking of the predetermined power and pressure set-points. Ferramosca et al.^13^ gave zone MPC, which can achieve zone tracking steady-state set-points in the target zone, however, zone MPC method has dynamic zone tracking errors. Zhang et al.^14^ described a zone economic MPC controller to optimize the operating economic in boiler-turbine system. Liu et al.^15^ steady state target optimization layer in RTO and MPC, which choose a set of steady-state operating setpoints in the cutting tobacco dying process.

This paper aims to design a priority multi-objective optimization strategy for system output variables based on the existing MPC strategy framework. The remainder of the paper is organized as follows. “Introduction” section  provides a detailed cutting tobacco production and modelling process. “Cigarette production processes and modelling” section, the Multi-Objective MPC(MOMPC) optimal algorithm is proposed. In “Modelling of the cutting tobacco drying process” section, MOMPC feasibility testing and soft constraint adjustment are presented. “The control algorithm of the cutting tobacco drying process” section gives the simulation of the multi-objective control strategy proposed above. “Simulation result of the multi-objective MPC control strategy” section presents the conclusion.

## Cigarette production processes and modelling

### The cigarette production process

**Figure 1:**
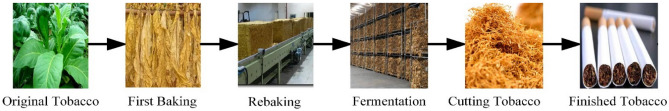
Production process of cigarette.

**Figure 2:**
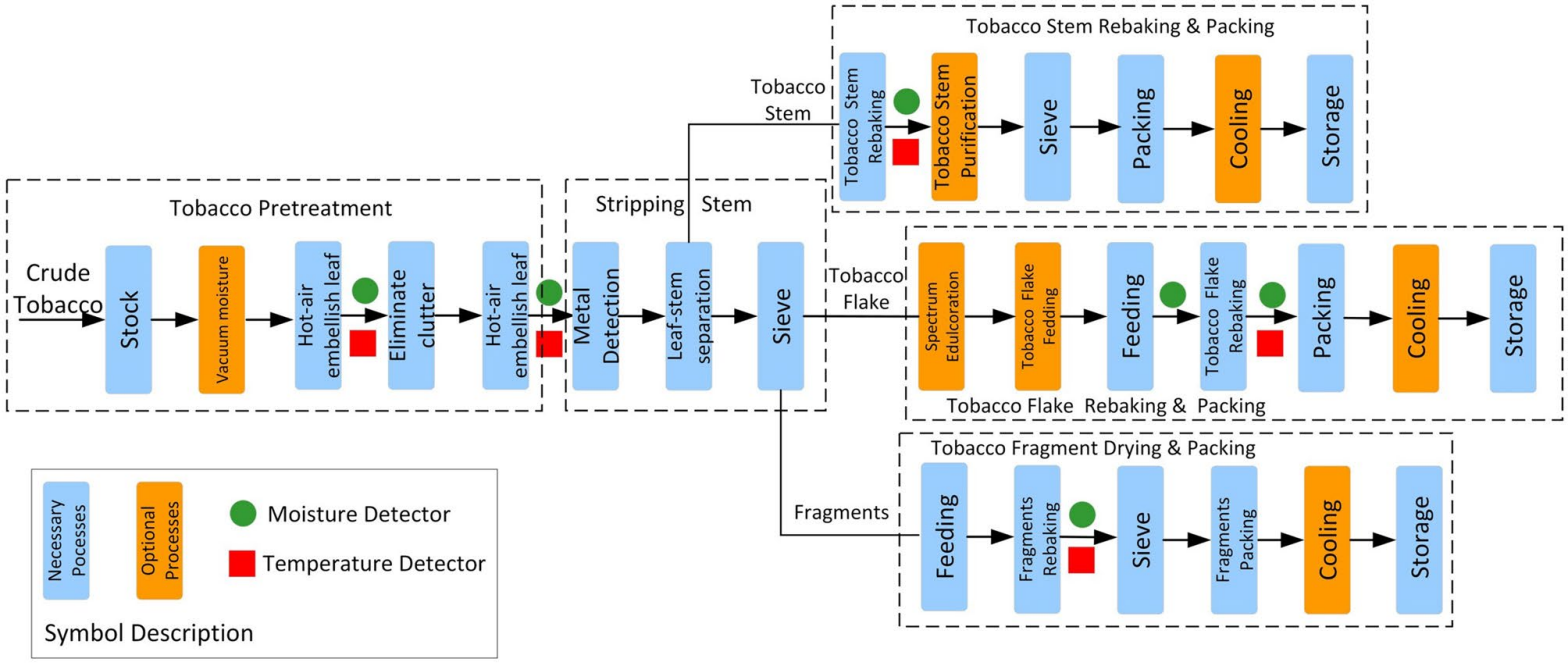
Industrial process flow chart of cigarette production.

The cigarette production process is a complex industrial process with a long working procedure and high control precision. The raw tobacco changed from yellowish green to yellow dry coke after embellish leaf roasting. To facilitate storage, the tobacco after roasting is separated into stems and leaves, and secondary moisture adjustment is carried out. The separated tobacco leaves are re-roasted to reach the required moisture content, and fermented to increase flavor and improve color and smell. The separated stems are re-roasted and then cut into pieces with tobacco leaves to form cigarette tobacco. The production process of finished tobacco cigarettes can be roughly described as shown in Fig. 1. The detailed industrial process flow chart of cigarette production is shown in Fig. 2.

The technological requirements and specifications index of the cigarette production process will be described in detail below. The cigarette production processes mainly include the following steps:

The first step is selection and preparation, and the technological requirements are to prepare raw tobacco, batches and stack them in order or in module ratios^16^.

The second step is to vacuum regain moisture and the technological requirements are to increase moisture content, raise tobacco leaf temperature, and loosen them^16,17^. The specifications index and requirement are listed in Table 1.

**Table 1:** Vacuum regain moisture index and requirement.

Index	Requirement
Inside core temperature	$\le 75^{ \circ } {\text{C}}$
Moisture content increase	$\ge 2.0$
Return permeability	$\ge 98.0$

The third step is the hot-air tobacco leaf moisture, and the technological requirements are to increase the moisture content and raise the tobacco leaf temperature through hot-air. The process ability resistance of tobacco leaves is improved^16,17^. The specifications index and requirement are listed in Table 2.

**Table 2:** Hot-air tobacco leaves moisture index and requirement.

Index	Requirement
Temperature	$(50 - 70)^{ \circ } {\text{C}}$
Moisture content	$(17 - 20)\%$
Allowable error of moisture content	$\pm 1\%$
Looseness rate	$\ge 99.0$

The fourth step is to separate leaves and stems, and the technological requirements are to separate and sieve tobacco leaves and stems. The structure of tobacco leaves is greater than > (12*.*7 mm × 12*.*7 mm) over 80%, and the stems are greater than > 20 mm over 85%^16^.

The fifth step is to re-bake tobacco flakes. The technological requirements are to dry, cool and re-wet the tobacco flakes, and the moisture content of the tobacco flakes is regulated for mellowing and storage. The specifications index and requirement are listed in Table 3.

**Table 3:** Tobacco flakes re-baking index and requirement.

Index	Requirement
Temperature	$(40 - 60)^{ \circ } {\text{C}}$
Moisture content	$(11 - 13.5)\%$
Standard deviation of moisture content	$\le 0.33\%$

The sixth step is to pack tobacco flakes, and the technological requirements are to package and bundle the re-baking tobacco flakes according to the packaging rules^16^. The specifications index and requirement are listed in Table 4.

**Table 4:** Tobacco flakes re-baking packing index and requirement.

Index	Requirement
Temperature	$(35 - 45)^{ \circ } {\text{C}}$
Moisture content	$(10.5 - 13.0)\%$

The seventh step is to re-bake the tobacco stems, and the technological requirements are to dry the tobacco flakes for easy storage^16,17^. The specifications index and requirement are listed in Table 5.

**Table 5:** Tobacco stem re-baking index and requirement.

Index	Requirement
Moisture content	$(10.0 - 13.0)\%$

The eighth step is to pack the tobacco stems^16^. The specifications index and requirement are listed in Table 6.

**Table 6:** Tobacco stems packing index and requirement.

Index	Requirement
Moisture content	$(10.0 - 13.0)\%$
Net weight allowance	$( \pm 0.5\,{\text{kg}}/{\text{ctn}})\%$

The ninth step is to re-bake the tobacco fragments. The technological requirements are to collect, dry and cool the tobacco fragments, and the moisture content is controlled for storage^16^. The specifications index and requirement are listed in Table 7.

**Table 7:** Tobacco fragment re-baking index and requirement.

Index	Requirement
Moisture content	$(11.0 - 13.0)\%$
Stem containing rate	$\le 0.2\%$

The tenth step is to cool tobacco after processing, and the technological requirements are to store the packaged tobacco flakes, tobacco stems, and tobacco fragments for a period of time to decrease the temperature^16^.

The last step is to cut tobacco into pieces. The technological requirements are to cut the tobacco leaves and stems into tobacco shreds through a shredder, and the moisture content and filling value can meet the technological production requirements. The cigarettes are finished^17^.

## Modelling of the cutting tobacco drying process

The main task of cutting tobacco drying is to control the outlet moisture, meanwhile the nonlinear mathematical model of cutting tobacco drying is established by using the first principle^18^. The opening degree of the air door and temperature are constantly adjusted according to the model to effectively control the moisture content of tobacco.

The drying process of cutting tobacco is related to the inlet flow of cutting tobacco, the inlet moisture of cutting tobacco, the rotational speed of the drying cylinder, the opening degree of the steam valve, the temperature and speed of hot air, the opening degree of the hot air damper, and the pressure and temperature of the steam in the drying cylinder^19^. The drying model of cutting tobacco was established based on the analysis of the influencing factors of the drying process. Assume that the drum tobacco machine is adiabatic (heat losses *Q*_*c*1_ and *Q*_*c*2_ are equal to 0). The drum length *L* is 7.7 m, the diameter *D* is 1.25 m, and the inclination angle of the drum tobacco machine is 3*.*5 °C^20^. The stripping of liquid water during drying should ensure that unnecessary biochemical reactions do not occur, that is, the thermal and chemical properties of materials, air and water are constant within the temperature range considered, and the drying air is evenly distributed in the dryer. The mass flow at the input and output of the drum tobacco machine is equal.

The moisture mass balance equation of cutting tobacco^21^ is established as follows:

1 $$\rho_{{{\text{tobacco}}}} V\frac{{d\omega_{{{\text{out}}}} }}{dt} = m_{{{\text{in}}}} \omega_{{{\text{in}}}} - m_{{{\text{out}}}} \omega_{{{\text{out}}}} - \rho_{{{\text{tobacco}}}} VR_{{{\text{cdr}}}}$$

Here $\rho_{{{\text{tobacco}}}}$ is the cutting tobacco density. $\omega_{{{\text{in}}}}$ is the cutting tobacco moisture content of the inlet. $\omega_{{{\text{out}}}}$ is the outlet cutting tobacco moisture content. $m_{{{\text{in}}}} = m_{{{\text{out}}}}$ is the quality of cutting tobacco. $R_{{{\text{cdr}}}}$ is the drying rate of cutting tobacco. $v$ is drum volume.

The dynamic energy balance equation of drum temperature^21^ is established as follows:

2 $$\begin{aligned} \frac{{dT_{{{\text{dryer}}}} }}{{d{\text{t}}}} & = \frac{{\rho_{{{\text{air}}}} c_{{{\text{hotair}}}} q_{{{\text{hotair}}}} (T_{{{\text{hotair}}}} - T_{{{\text{dryer}}}} )}}{{\rho_{{{\text{mix}}}} Vc_{{{\text{mix}}}} }} \\ & \quad + \frac{{m_{{{\text{in}}}} c_{{{\text{tobacco}}}} (T_{{{\text{int}}}} - T_{{{\text{outt}}}} )}}{{\rho_{{{\text{mix}}}} Vc_{{{\text{mix}}}} }} \\ & \quad + \frac{{\rho_{{{\text{tobacco}}}} VR_{{{\text{cdr}}}} c_{{{\text{lw}}}} (T_{{{\text{int}}}} - T_{{\text{f}}} )}}{{\rho_{{{\text{mix}}}} Vc_{{{\text{mix}}}} }} \\ & \quad + \frac{{AH_{{0}} (T_{{{\text{c2}}}} - T_{{{\text{dryer}}}} )}}{{L\rho_{{{\text{mix}}}} Vc_{{{\text{mix}}}} }} - Q_{{{\text{l2}}}} \\ \end{aligned}$$

Here $T_{{{\text{dryer}}}}$ is the drum temperature. $\rho_{{{\text{air}}}}$ is the air density. $c_{{{\text{hotair}}}}$ is the hot air thermal capacity.$T_{{{\text{air}}}}$ is the hot air temperature. $q_{{{\text{hotair}}}}$ is the hot air volume flow.$c_{{{\text{tobacco}}}}$ is the cutting-tobacco thermal capacity. $T_{{{\text{int}}}}$ is the inlet cut tobacco temperature. $T_{{{\text{outt}}}}$ is the outlet cut tobacco temperature. $\rho_{{{\text{mix}}}}$ is the mixture density of drum. $c_{{{\text{mix}}}}$ is the mixture thermal capacity of the drum. $c_{{{\text{lw}}}}$ is the liquid water thermal capacity.

The dynamic energy balance equation of the cutting tobacco outlet temperature^21^ is established as follows:

3 $$\frac{{dT_{{{\text{outt}}}} }}{{d{\text{t}}}} \, = \frac{{H_{{1}} (T_{{{\text{dryer}}}} - T_{{{\text{outt}}}} ) + H_{{1}} (T_{{{\text{hotair}}}} - T_{{{\text{outt}}}} )}}{{L\rho_{{{\text{tobacco}}}} Vc_{{{\text{tobacco}}}} }} - \frac{{\rho_{{{\text{tobacco}}}} VR_{{{\text{cdr}}}} c_{{{\text{lw}}}} (T_{{{\text{outt}}}} - T_{{{\text{int}}}} )}}{{\rho_{{{\text{tobacco}}}} Vc_{{{\text{tobacco}}}} }}$$

The dynamic energy balance equation of hot air temperature^21^ is established as follows:

4 $$\frac{{dT_{{{\text{hotair}}}} }}{{d{\text{t}}}} = \frac{{\rho_{{{\text{air}}}} c_{{{\text{hotair}}}} q_{{{\text{hotair}}}} (T_{{{\text{in}}}} - T_{{1}} )}}{{\rho_{{{\text{aw}}}} Vc_{{{\text{tobacco}}}} }} + \frac{{H_{{2}} (T_{{{\text{c1}}}} - T_{{{\text{hotair}}}} )}}{{L\rho_{{{\text{aw}}}} Vc_{{{\text{aw}}}} }} - Q_{{{\text{l1}}}}$$

Here $T_{{{\text{in}}}}$ is the inlet air temperature. $H_{{0}} ,H_{{1}} ,H_{{2}}$ are the coefficients of thermal conductivity, $H_{{0}} = 100({\text{W}}/{\text{m}}^{ \circ } {\text{C}}),H_{{1}} = 5({\text{W}}/{\text{m}}^{ \circ } {\text{C}}),H_{{2}} = 700({\text{W}}/{\text{m}}^{ \circ } {\text{C}})$. $T_{{\text{f}}}$ is the indoor reference temperature. $T_{{{\text{c1}}}}$ is the steam temperature of the heater. $T_{{{\text{c2}}}}$ is the heating steam temperature of the drum. $\rho_{{{\text{aw}}}}$ is the mixture density of the hot dryer. $C_{{{\text{aw}}}}$ is the mixture thermal capacity of the hot dryer. $Q_{{{\text{l1}}}} ,Q_{{{\text{l2}}}}$ is the heat loss.

According to the modelling process of cutting tobacco drying, the drying process model is a nonlinear and non-square model^22^. The controlled output variables of the system are the outlet cutting tobacco moisture content $\omega_{{{\text{out}}}}$, drum temperature $T_{{{\text{dryer}}}}$, hot air temperature $T_{{{\text{dryer}}}}$ and outlet cutting tobacco temperature $T_{{{\text{outt}}}}$. The operational input variable is the two-way steam temperature *T*_c1_ and *T*_c2_. For the cutting tobacco drying process, the most critical controlled output variable (outlet cutting tobacco moisture) is mainly affected by the temperature of the drying cylinder and the hot air temperature. The number of controlled output variables of the drying process model is greater than the number of operating input variables, which puts the control system in a weak control state (insufficient system control freedom)^23^. To enable the system to operate strictly under the process requirements, this paper carries out priority ascending optimization for the controlled output variables of the system, and then softens the priority descending order of additional target constraints when optimizing the control of specific output variables. The first step is to relax the target constraint interval with low priority.

## The control algorithm of the cutting tobacco drying process

The Multi-Objective Optimization (MOO) strategy algorithm has been widely used in optimal control systems for a long time. Molina et al. and Rani et al.^24,25^ presented using a simplified goal of the MOO problem to realize the adjustment of PI and PID controllers based on MOO design. Reynoso et al.^26^ developed the design of a Two-Degree-of-Freedom (2-DoF) robust PID controller based on the partial model matching method. Gatzke et al.^27^ proposed the MOO control framework of MPC, which can be used to sort and control the controlled output targets of the system according to preset priorities based on a dictionary sorting algorithm. Wojsznis et al.^28^ constructed another MOO-MPC control strategy, in which the performance index is expressed as a MOO optimization problem, and the optimal operation input variables are solved by the goal attainment method.

### Basis of the multi-objective model predictive control optimization algorithm

To achieve the optimal operation of the system-controlled output variables, the classical control PID and related non-optimized control strategies are usually adjusted according to engineering experience, and the related intelligent optimization strategies (such as neural networks, ant colony algorithms and multi-objective algorithms) can also be used to adjust the PID parameters, but the final effect is limited^29^. The control strength of the non-optimized strategy is often only related to the linear model. For complex nonlinear models, MPC is more commonly used in engineering. To obtain the optimal control effect, there are many algorithms and strategies to optimize the parameters of the MPC control framework. The existing MPC tuning methods are generally divided into two categories^30,31^. The first method is to obtain analytical expressions by simplifying the process description or process model to some extent, and to add some parameter adjustments. The relevant performance indicators are combined into the overall adjustment objective function based on the technology of multi-objective optimization, according to the definition of the goal, and the use of a multi-objective optimization algorithm to solve the tuning problem of the technology is different. These methods have different regulation objective definitions, time domain characteristics (such as regulation time, rise time, overshoot), time domain performance indicators (such as square error integral), frequency domain sensitivity norms, and related combinations of different objective functions^32–34^. The MPC adjustment method takes the minimum error between the closed-loop response and the output variable reference trajectory as the objective function. The second method is to sort the output variables according to the importance of the controlled output variables to the process operation, and solve them by a dictionary optimization algorithm^35^. For general nonlinear systems, the MPC objective function includes the weighted sum of the square deviation between the predicted output and the set value in the prediction time domain, and the weighted sum of the square input increment in the control time domain. The MPC control problem is shown as follows:

5 $$\begin{aligned} \mathop {\max }\limits_{{\Delta u_{k} }} V_{1} & = \sum\limits_{j = 0}^{p} {\left\| {y(\left. {k + j} \right|k) - y_{sp} } \right\|_{{Q_{y} }}^{2} } + \sum\limits_{j = 0}^{m - 1} \left\| {\Delta u(\left. {k + j} \right|k)} \right\|_{R}^{2} \\ s.t.\quad 0 & = f\left( {\frac{dx}{{dt}}, \, x, \, y, \, p, \, d, \, y, \, u} \right) \\ u_{\min } & \le u(k + j) \le u_{\max } ,\quad j = 0, \ldots ,m - 1 \\ \Delta u_{\max } & \le \Delta u(k + j) \le \Delta u_{\max } ,\quad j = 0, \ldots ,m - 1 \\ \end{aligned}$$

The multi-objective optimization strategy has two main trade-off choices to handle competing objectives, appropriately weighting the objectives before solving the problem or selecting the optimal solution according to the subjective criteria after obtaining a set of optimal solutions. The general multi-objective problem is expressed as follows:

6 $$\begin{aligned} & \mathop {\min }\limits_{x} F(x) = \left[ {\begin{array}{*{20}l} {F_{1} (x)} \hfill & {F_{2} (x)} \hfill & L \hfill & {F_{w} (x)} \hfill \\ \end{array} } \right]^{{\text{T}}} \, \\ & s.t.\quad g_{j} (x) \le 0,j = 1, \ldots ,z \\ & h_{l} (x) = 0,l = 1, \ldots ,e \, \\ \end{aligned}$$

Here $F(x)$ is a vector composed of $\omega$ objective functions $F_{i} , \, g_{j} (x)$ and $\, h_{l} (x)$ are the system inequality and equality constraints, respectively. $X = \{ x \in R^{n} |g_{{\text{j}}} (x) \le 0,j = 1, \ldots ,z,h_{{\text{l}}} (x) = 1, \ldots ,e\}$ is the feasible region space. $n_{{{\text{dec}}}}$ is the vector of optimization decision variables. $n$ is the number of decision variables. $Z = \left\{ {z \in R^{w} \left| {z = F(x),x \in X} \right.} \right\}$ is the feasible criterion space. $F_{{\text{i}}} (x)$ is defined according to preferences or economic goals imposed by decision-makers.

In the MPC control strategies of many process systems, the importance of controlled output variables can be sorted. The dictionary target sorting algorithm is used to tune the controller to establish the optimization problem of the hierarchical control system^36^. Assume that the output variable targets and priority rankings are defined by the operator in this paper. In each step, optimization problems can be split into many single-objective optimization problems to be solved, each of which is solved in the order of importance^37^. In each optimization step, the previously obtained optimal cost function value is included as a constraint in the new optimization problem.

The objective function is sorted by importance based on dictionary optimization technology^38^. This method is suitable for step response in the finite output time domain and state space response in the infinite output time domain. First, the operator needs to analyze the relative importance of the process -controlled output variables, usually taking economic, security and environmental factors as guidelines of control strategies. Second, an input–output pair is defined for each process -controlled output variable according to the importance order of the controlled output variables. Third, the values and benefits of the input and output variables of the system model are normalized, and the purpose is to optimize the value of the tuning cost function of different objectives on a similar order of magnitude. Fourth, the error between the output closed-loop response and the reference trajectory is adjusted to be the smallest. The operator can define the time constant of the objective function according to the order of importance of the output variable and the specifications of the process operator^39–41^. The MOO of the output variable target is defined as,

7 $$F_{{\text{i}}} (x) = \sum\limits_{k = 1}^{{\theta_{{\text{t}}} }} {\left( {y_{{\text{i}}}^{{{\text{ref}}}} (k) - y_{{\text{i}}} (k)} \right)^{2} } ,\quad i = 1, \ldots ,w$$

Here $\theta_{{\text{t}}}$ is the time domain adjustment. $y_{i}^{{{\text{ref}}}} (k)$ is the reference trajectory for discretization of controlled output variable $i$, $y_{{\text{i}}} (k)$ is the closed loop trajectory of the controlled output variable $i$ and $x$ is a vector of decision variables or tuning parameters. $w$ is the number of input–output pairs. $Q_{{\text{y}}} = diag(q_{1} , \ldots ,q_{{{\text{ny}}}} )$, $R = diag(r_{1} , \ldots ,r_{{{\text{nu}}}} )$ are diagonal weight matrices. $x = (q_{1} , \ldots ,q_{{{\text{ny}}}} ,r_{1} , \ldots ,r_{{{\text{nu}}}} )$*.*$y_{{\text{i}}} (k)$ is the response of the closed-loop. The optimal control input variables are obtained by minimizing Eq. (7). The importance of process-controlled output variables also represents the dictionary optimization order. The definition of the MOMPC optimization problem is shown as follows:

8 $$\begin{aligned} & \mathop {\min }\limits_{\Delta u,\delta } \;V_{2} = \sum\limits_{i = 1}^{{w^{\prime } }} {F_{{\text{i}}} (x)} + \delta^{T} S_{{\text{t}}} \delta \\ & s.t.\;F_{{\text{i}}} (x) = \sum\limits_{k = 1}^{{\theta_{{\text{t}}} }} {\left( {y_{{\text{i}}}^{{{\text{ref}}}} (k) - y_{{\text{i}}} (k)} \right)^{2} } ,\;i = 1, \ldots ,w \\ & F_{{\text{i}}} (x) - F_{{\text{i}}}^{*} - \delta_{{\text{i}}} \le 0,\;i = 1, \ldots ,w^{\prime } - 1 \\ & \delta_{{\text{i}}} \ge 0,\;i = 1, \ldots ,w^{\prime } - 1 \\ & y_{{{\text{LL}}}} \le y \le y_{{{\text{UL}}}} \\ & u_{{{\text{LL}}}} \le u_{{\text{i - 1}}} + \Delta u \le u_{{{\text{UL}}}} \\ \end{aligned}$$

Here $i$ is the current tuning step. $w^{\prime }$ is the number of current output targets, $\delta$ is the vector of the relaxation variable, and $S_{{\text{t}}} \in R^{{(w^{\prime } - 1) \times (w\prime - 1)}}$ is the diagonally weighted matrix. $y_{{{\text{LL}}}}$ and $y_{{{\text{UL}}}}$ are the lower and upper bounds of the decision variables. When dealing with lower priority output targets, the goal defined in the multi-objective optimization attempts to force higher priority output variables to be prioritized to obtain the best performance. Relaxation variable $\delta$ ensures that multi-objective optimization problems are always feasible. $F_{{\text{i}}}^{*}$ is the optimal value of variable $y_{{\text{i}}}$ in the $ith$ dictionary priority target.

The purpose of multi-objective optimization is to find a decision variable or parameter vector that satisfies the constraint conditions, and optimize the vector space, whose spatial elements represent the objective function. This method can improve the feasibility of the MPC control strategy by relaxing the constraints according to the online assignable priority.

### Feasibility testing and soft constraint adjustment of multi-objective MPC

If the operation and engineering constraints of the system cannot form an effective feasible region, the MOMPC optimization results cannot be obtained. The optimization strategy can be implemented only when the feasible region of the system exists. Drying and other industrial processes, are not allowed to interrupt the control strategy, because the infeasible regions in the production process will affect the quality of the production process and the safety of the plant operation^42^. For the multi-objective MPC control strategy, a feasible region testing mechanism and optimization implementation are necessary to ensure the smooth implementation of the control strategy.

The feasible region testing mechanism mainly aims at whether there is an effective feasible region in the constrained region of the system before optimization, so that the optimal solution can be found in the optimization strategy^43^. If the system has infeasible regions, some constraints must be adjusted to make the constraint space have feasible regions. The system is mainly subject to two types of constraints, hard constraints and soft constraints. Generally, the hard constraint is the constraint of the input variable of system operation (the physical constraint cannot be violated), and the soft constraint is the constraint bound of the output variable controlled by the system (operation constraint and engineering constraint). The operation constraint boundary is $y_{{{\text{LL}}}} \le y \le y_{{{\text{UL}}}}$, and the engineering constraint boundary is $y_{{{\text{LLL}}}} \le y \le y_{{{\text{UUL}}}}$. The engineering constraint is a hard constraint for the controlled output variable. The infeasible region solution is constraint adjustment, i.e., soft constraint adjustment. The constraints of the controlled output variables of the system are appropriately relaxed, but the soft constraints must not be relaxed beyond the engineering constraints. When the feasible region does not require softening constraints, the feasible region of the system is shown as follows:

9 $$\begin{aligned} & 0 = f\left( {\frac{dx}{{dt}},x,y,p,d,u} \right) \\ & u_{{{\text{LL}}}} \le u_{{\text{i - 1}}} + \Delta u \le u_{{{\text{UL}}}} \\ & y_{{{\text{LL}}}} \le y \le y_{{{\text{UL}}}} \\ \end{aligned}$$

When the feasible region does not exist, the relaxation variables are introduced for the constraint of the controlled output variable, which is shown as follows:

10 $$\begin{aligned} & 0 = f\left( {\frac{dx}{{dt}},x,y,p,d,u} \right) \\ & u_{{{\text{LL}}}} \le u_{{\text{i - 1}}} + \Delta u \le u_{{{\text{UL}}}} \\ & y_{{{\text{LL}}}} - \varepsilon_{1} \le y \le y_{{{\text{UL}}}} + \varepsilon_{2} \\ & y_{{{\text{LLL}}}} \le y \le y_{{{\text{UUL}}}} \\ \end{aligned}$$

Here $\varepsilon_{1}$ and $\varepsilon_{2}$ are relaxation variables of the controlled output variables constrained, and the constraints without relaxation variables are hard constraints.

The MOMPC control strategy optimization implementation stage mainly seeks an effective optimal solution in the effective feasible region when there is an effective feasible region. However, the feasible region cannot guarantee the optimality of the optimal target solution, which may cause the target to deviate from the expected target value. In the feasible region, the system can be adjusted to drive to the expected target in the feasible region through an adjustable residual freedom constraint, and the optimal solution can be obtained. The feasibility determination of the optimization problem and the weighting method of soft constraint adjustment of input and output variables can uniquely determine the optimization feasible region. If there is an optimization feasible region, the optimal solution of the optimization target can be found in the feasible region space in the optimization implementation stage^44–46^.

First, the feasibility problem of MOMPC control strategy optimization was determined, that is, the feasibility of the optimization problem was determined according to the nonlinear model and constraints of the industrial process. Then, the soft constraint is adjusted, that is, the constraint boundary is relaxed to make the optimization problem feasible when the optimization result is judged to be infeasible. For simple constraints, the graphical method is used to determine whether the optimization problem is feasible. However, for the general multi-objective MPC control strategy optimization problem, which involves a nonlinear process and constraint conditions, the feasibility testing problem is more merged into a soft constraint adjustment problem. If the optimal solution of the decision variable is zero, there is a feasible region in the constraint space of the process. If the decision variable is a non-zero solution, that is, the constraint space needs the decision variable of a non-zero solution to obtain the optimization feasible region. If the optimization has no solution, the constraint feasible region cannot be obtained by relaxing the variables. Then, the constraint feasible region needs to be reconstructed by region relaxation of the target trajectory.

### Multi-objective priority and objective constraint priority adjustment

In real industry, the importance of each controlled output variable of the system is inconsistent, and it is necessary to distinguish the priority of the output variable to better optimize the control. For the cutting tobacco drying process, the non-square model has an insufficient control degree of freedom, which leads to the steady-state error of the conventional control strategy. Therefore, the priority control strategy of controlled output variables is adopted^47^. In the drying process of cutting tobacco, the outlet moisture content $\omega_{cs}$ is the most critical controlled output variable of the system, and it should be given priority to achieve the optimal control state in accordance with various constraints of the system. The multi-objective priority control strategy is based on the MPC control framework, and the objective priority is adopted to optimize the control of output variables in a certain order. The priority of the controlled output variable of the system represents the importance of the output variable, and the higher the priority, the more critical it is.

The controlled output variables of the real industrial process are also subject to additional objective constraints, economic objective constraints, safety objective constraints, and ecological environment objective constraints. When the feasible region determined by the operation and engineering constraints of the controlled output variables is satisfied, the system needs to determine the priority order of the target constraints on each controlled output variable after determining the priority order of each controlled output variable, so that the controlled output variables run along the optimal target trajectory. Assuming that each output variable has $r_{n}$ priorities,

11 $$\left\{ {\begin{array}{*{20}c} {\xi^{r} \varepsilon^{r} = b_{1}^{r} } \\ {\Phi^{r} \varepsilon^{r} \le b_{2}^{r} } \\ \end{array} } \right.,\;r = 1, \ldots ,r_{n}$$

Here $\xi$ and $b_{1}$ are the system parameters of equality target constraints. $\Phi$ and $b_{2}$ are the system parameters of non-equality target constraints. $r$ is the target constraint priority series of the current controlled output variables. The target constraint is as follows when $r = 1$.

In the multi-objective priority control strategy, when the control strategy exists in the feasible region of the system, the controlled output variables of the system are first optimized by priority ascending order, and when the specific output variables, the priority of the additional target constraints is softened in descending order, and the target constraints with low priority are relaxed first^48^. The specific control strategy is divided into two stages. ① The system-controlled output variables are prioritized in ascending order, and the corresponding weight coefficients are set. The technological requirements of the system -controlled output variables with the highest priority are first met. ② After the priority of the controlled output variable is determined, the target constraint descending priority and weight coefficient of the specific controlled output variable are determined. When the target constraint has r priorities, if the target constraint interval is not feasible, the target priority with *r* priority will be relaxed first, and then the priority of ($r = 1$) will be optimized. If the target constraint interval is feasible, the target constraint of other priorities will no longer be optimized.

A feasible region of system constraint exists or the feasible region exists through soft constraint adjustment. The multi-objective priority control strategy first considers the priority ascending order of the controlled output variables of the system to determine the importance of the controlled output variables of the system. For the additional objective constraint, descending order is carried out to obtain the optimal trajectory of the controlled output variables. The multi-objective optimization control strategy of cutting the tobacco process is shown as follows:

12 $$\begin{aligned} & \min {\text{V}}_{3} = \sum\limits_{i = 1}^{{\omega^{\prime}}} \sum\limits_{k = 1}^{{\theta_{t} }} \left\{ {\left[ {\begin{array}{*{20}l} {\omega_{{\text{out }}}^{{\text{ref }}} (k)} \hfill \\ {T_{{{\text{dryer}}}}^{{{\text{ref}}}} (k)} \hfill \\ {T_{{{\text{out}}}}^{{{\text{ref}}}} (k)} \hfill \\ {T_{{{\text{hotair}}}}^{{{\text{ref}}}} (k)} \hfill \\ \end{array} } \right] - \left[ {\begin{array}{*{20}c} {\omega_{{\text{out }}} (k)} \\ {T_{{\text{dryer }}} (k)} \\ {T_{{\text{outt }}} (k)} \\ {T_{{\text{hotair }}} (k)} \\ \end{array} } \right]} \right\}^{2} \\ & \quad + \left[ {\delta_{1} \delta_{2} \ldots \delta_{i} \ldots \delta_{n} } \right]{\text{diag}} \left( {r_{1} r_{2} \ldots r_{{{\text{mu}}}} } \right)\left[ {\begin{array}{*{20}l} {\delta_{1} } \hfill & {\delta_{2} } \hfill & { \ldots } \hfill & {\delta_{{\text{n}}} } \hfill \\ \end{array} } \right]^{T} \\ & \quad + \left( {\varepsilon^{{r_{{\text{n}}} }} } \right)^{T} \left( {W_{{\text{Q}}}^{{r_{{\text{n}}} }} } \right)^{2} \varepsilon^{{r_{{\text{n}}} }} \\ & {\text{s}}{\text{.t }}\quad x = \left[ {\begin{array}{*{20}c} {T_{c1} } \\ {T_{c2} } \\ \end{array} } \right] \\ & \left[ {\begin{array}{*{20}l} {\omega_{{\text{out }}}^{{\text{ref }}} (k)} \hfill \\ {T_{{\text{dryer }}}^{{\text{ref }}} (k)} \hfill \\ {T_{{\text{outt }}}^{{\text{ref }}} (k)} \hfill \\ {T_{{\text{hotair }}}^{{\text{ref }}} (k)} \hfill \\ \end{array} } \right] - \left[ {\begin{array}{*{20}l} {\omega_{{\text{out }}} (k)} \hfill \\ {T_{{\text{dryer }}} (k)} \hfill \\ {T_{{\text{outt }}} (k)} \hfill \\ {T_{{\text{hotair }}} (k)} \hfill \\ \end{array} } \right] - \delta_{i} \le 0\quad i = 1, \cdots ,\omega^{\prime } - 1 \\ & \delta_{i} \ge 0\quad \quad i = 1, \ldots ,(\omega^{\prime } - 1) \\ & \mathop \prod \limits^{{r_{n} }} \varepsilon^{{r_{n} }} = b_{1}^{{r_{n} }} \\ & \Phi^{{r_{n} }} \varepsilon^{{r_{n} }} \le b_{2}^{{r_{n} }} \% \\ \end{aligned}$$

Here $W_{{\text{Q}}}^{{r_{0} }}$ is the positive-definite weight coefficient matrix. Because it is a descending-order softening target constraint, only softening and relaxing the target constraint corresponding to the minimum priority $r_{n}$ are considered, and other priority target constraints are treated as hard target constraints. The multi-objective optimal control strategy based on MPC can prioritize the controlled output variables online when the system does not have sufficient degrees of freedom to satisfy the process requirements of the controlled output variables, so that the system can prioritize meeting the process requirements of the controlled output variables to alleviate the problem of insufficient degrees of freedom of nonlinear systems. The controlled output variable may also be subject to additional artificial optimization target constraints, which can be further assigned to the target constraints in descending order after the controlled output variable’s priority has been determined, so that the controlled output variable always runs within the optimal target trajectory, which improves the feasibility and accuracy of the control system.

## Simulation result of the multi-objective MPC control strategy

### Verification of the multi-objective control strategy for a multi-variable system

For the multi-variable system, each set value optimization of the system-controlled output variable has a mutual coupling effect, especially in the non-square model of the tobacco drying process. When the number of input variables is insufficient, the priority of the controlled output variables is the key to the system control strategy. The model predictive control of priority multi-objective and soft constraint weighting adjustment is used to control and adjust the drying process of tobacco, so that it can meet the relevant technological requirements. The tobacco drying process model is a fourth-order nonlinear multi-variable model, and the four output variables of the tobacco drying process reach different target setting values. The outlet moisture content of cutting tobacco is the most critical output target of the drying process with the highest priority. The drum temperature of the cutting tobacco drying process has a higher priority. Hot air temperature and cutting tobacco outlet temperature have the lowest priority.

The priority multi-objective control strategy is compared with the traditional industrial predictive control strategy. The control strategy is divided into two scenarios. In Scenario 1, the performance of the two control strategies is compared under the nominal condition of the cutting tobacco drying process. The priority is absent from the traditional predictive control strategy. In the case of insufficient operating input variables, the control system will only meet the controlled output variables directly related to the operation input variables, but not the key system-controlled output variables. The multi-objective control strategy adds priority to make the target tracking optimization of the controlled output variable have priority, especially when the operation input variable is insufficient, and the operation input variable is forced to satisfy the controlled output variable with the highest priority first. For ease of calculation and testing, the following assumes are given,

1. The roller dryer is assumed to be adiabatic;
2. Material, air, moisture is constant over the temperature range considered;
3. Dry air is evenly distributed inside the drying cylinder;
4. The mass flow at the input and output of the roller wire dryer must be equal;
5. The speed of tobacco and hot air, the specific heat of tobacco, water and air, the quality of cutting tobacco and hot air are always keep same.

The simulation parameters are listed in Table 8^49^.

**Table 8:** The parameters of MOMPC.

Parameter	Value
Mass flow of cutting tobacco (${\text{kg}}/{\text{min}}$)	500
Volume flow of dry air (${\text{m}}^{3} /{\text{min}}$)	2000/60
Specific heat of water liquid (${\text{KJ}}/{\text{kg}}^{^\circ } {\text{C}}$)	4.18
Specific heat of water vapor (${\text{KJ}}/{\text{kg}}^{^\circ } {\text{C}}$)	1.85
Specific heat of dry air (${\text{KJ}}/{\text{kg}}^{^\circ } {\text{C}}$)	1.01
Specific heat of the cutting tobacco (${\text{KJ}}/{\text{kg}}^{^\circ } {\text{C}}$)	1.83
Density of water (${\text{kg}}/{\text{m}}^{3}$)	1000
Density of the air (${\text{kg}}/{\text{m}}^{3}$)	1.293
Density of the cutting tobacco (${\text{kg}}/{\text{m}}^{3}$)	320
Inlet moisture content of the cutting tobacco	0.19
Outlet moisture content of the cutting tobacco	0.13–0.09
Inlet temperature of the cutting tobacco ($^{ \circ } {\text{C}}$)	30
Outlet temperature of the cutting tobacco ($^{ \circ } {\text{C}}$)	20–100
Hot air temperature ($^{ \circ } {\text{C}}$)	100–120
Temperature of drum dryer ($^{ \circ } {\text{C}}$)	130–170

**Figure 3:**
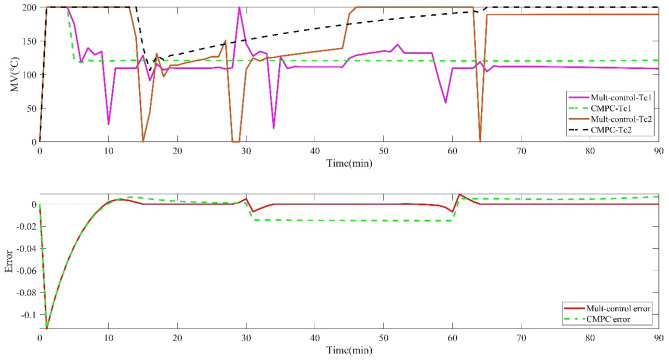
Operation variables of drying process and outlet moisture error of cutting-tobacco under nominal conditions.

**Figure 4:**
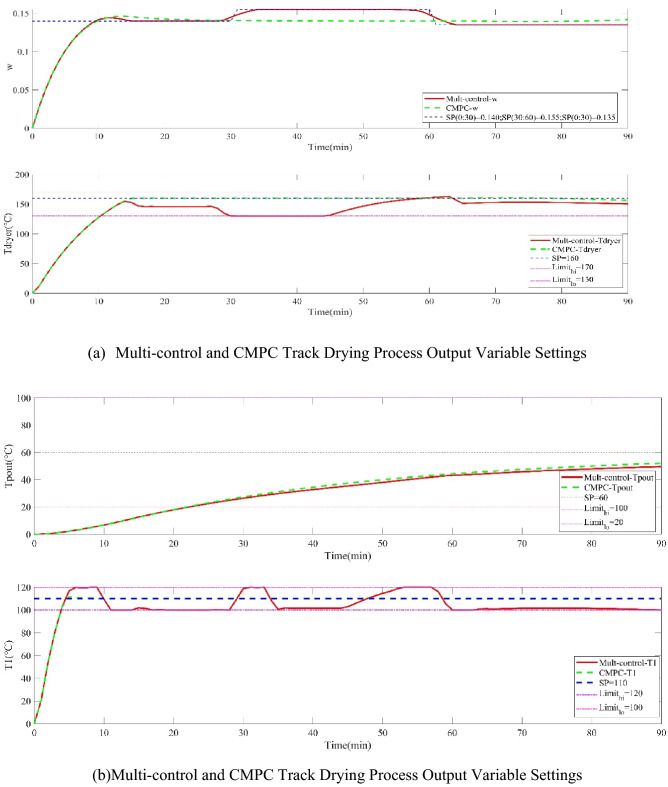
Multi-control and CMPC tracking optimization of tobacco drying process under nominal conditions.

The comparison is illustrated in Figs. 3 and 4.

Figures 3 and 4 show the operation input variables, the outlet moisture error of cutting tobacco and the tracking setting value of controlled output variables. When the Industrial Traditional Predictive Control Strategy (CMPC) is adopted, the two operation input variables of the system are directly related to the drum and hot air temperature. If the system freedom is insufficient, the controlled output variables of the two systems can only be satisfied, and the outlet moisture of cutting tobacco cannot be directly controlled, thus, there is a steady-state error. The priority multi-objective control strategy is adopted to make the outlet moisture of cutting tobacco have the highest priority. The system was forced to give priority to the outlet moisture set value of cutting tobacco under the system constraint, and the priority of the other three controlled output variables was reduced.

**Figure 5:**
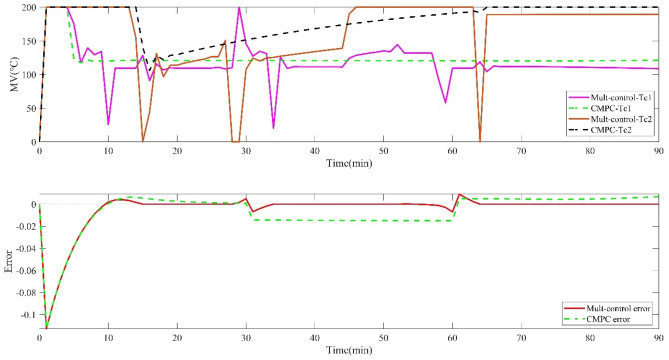
Operation variables of drying process and outlet moisture error of cutting tobacco under disturbance conditions.

**Figure 6:**
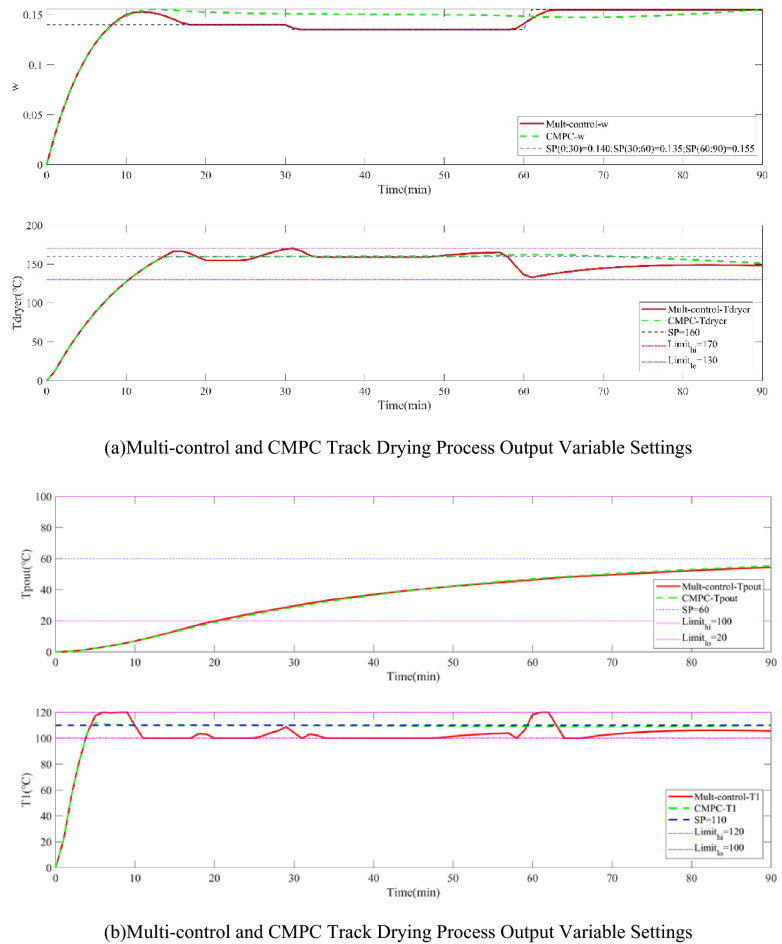
Multi-control and CMPC tracking optimization of tobacco drying process under disturbance conditions.

Scenario 2, The performance of the two control strategies is compared under the condition of disturbance existing in the tobacco drying process, which is shown in Figs. 5 and 6 which show the operation input variables, the outlet moisture error of cutting tobacco and the tracking setting value of the controlled output variables. Due to the lack of priority, traditional predictive control cannot meet the control requirements of the system in the case of disturbance when the degree of freedom is insufficient, while the multi-objective control strategy has good robustness when there is disturbance.

## Conclusion

In the drying process of cutting tobacco based on a nonlinear multi-variable model, mutual couplings exist in the system-controlled output variables. Since the control system is a non-square system with insufficient degrees of freedom, how to preferentially satisfy the system under the limited operation input variables is the most critical problem. Multi-objective optimization is an effective strategy when there is competition among objectives. The multi-objective optimization algorithm is introduced into the MPC control strategy, and the system-controlled output variables are optimized in ascending order of priority to meet the technological requirements of the controlled output variables with higher priority. When additional target constraints may exist in the controlled output variables, the target constraints of the controlled output variables are prioritized after determining the priority of the specific controlled output variables. The target constraints with low priority are first relaxed. The relaxing of other high-priority target constraints is stopped when the optimization is feasible. Eventually, the system-controlled output variables move along the optimal target trajectory to achieve the optimal control strategy.

## Data Availability

The data used to support the findings of this study are available from the corresponding author upon request.
